# LSD1 and HY5 antagonistically regulate red light induced-programmed cell death in *Arabidopsis*

**DOI:** 10.3389/fpls.2015.00292

**Published:** 2015-05-05

**Authors:** Tingting Chai, Jun Zhou, Jian Liu, Da Xing

**Affiliations:** MOE Key Laboratory of Laser Life Science and Institute of Laser Life Science, College of Biophotonics, South China Normal UniversityGuangzhou, China

**Keywords:** Arabidopsis, excess red light, EDS1, HY5, LSD1, programmed cell death, ROS, SA

## Abstract

Programmed cell death (PCD) in plant is triggered by abiotic and biotic stress. Light-dependent PCD is unique to plants. Light-induced PCD also requires reactive oxygen species (ROS) and salicylic acid (SA). In this study, lesion simulating disease1 (LSD1) and elongated hypocotyl 5 (HY5) perform opposite roles to regulate excess red light (RL)-triggered PCD associated with ROS and SA production. Under RL, the *lsd1* mutant released more ROS and SA and displayed a stronger cell death rate than the *hy5* mutant. It was shown that active LSD1 converted into inactive form by changing the redox status of the plastoquinone pool, and HY5 interacted with phytochrome B (phyB) to promote PCD in response to RL. LSD1 inhibited the enhanced disease susceptibility 1 (*EDS1*) expression by upregulating SR1, whereas HY5 enhanced the enhanced *EDS1* expression by binding to the G-box of the *EDS1* promoter. This study suggested that LSD1 and HY5 antagonistically modulated EDS1-dependent ROS and SA signaling; thus, PCD was mediated in response to RL.

## Introduction

Programmed cell death (PCD) is involved in plant growth and development. PCD is also closely related to defense during plant–pathogen interactions or acclimation in response to abiotic stress (Rusterucci et al., 2001; Gechev et al., 2006). The onset of PCD in plants is dependent on light, which is essential to the life cycle of plants. Excess light (EL) stress triggers the release of reactive oxygen species (ROS) and subsequently induces PCD in plants; red light (RL) is vital in this process (Asada, 1999; Mateo et al., 2004). ROS, which often interacts with salicylic acid (SA) and ethylene (ET), contributes to the progress of light-dependent PCD (Mazel and Levine, 2001; Muhlenbock et al., 2008; Li et al., 2013). RL-induced PCD is controlled partly by specific changes in redox status of the photosynthetic electron carrier chain, namely, plastoquinone (PQ) pool (Muhlenbock et al., 2008). Meanwhile, phytochrome B (phyB) is a major RL photoreceptor that positively regulates ROS production and cell death during de-etiolation response (Nagatani, 2004; Chen et al., 2013).

The phytochrome family (phyA–phyE) perceives red light (660 nm) and far-red (750 nm) light; this family also monitors light quality and intensity to regulate plant growth and development, such as seed germination, seedling de-etiolation, stem elongation, phototropism, stomata and chloroplast movement, shade avoidance, and flowering time (Franklin and Quail, 2010; Strasser et al., 2010). Phytochromes exist in two different photoreversible forms: RL-absorbing (Pr) and far-RL-absorbing (Pfr) forms. An inactive Pr is converted to an active Pfr; upon RL exposure, the active Pfr is translocated into the nucleus (Nagatani, 2004). Phytochrome-mediated light signals are dependent on SA signaling by regulating the expression of the enhanced disease susceptibility 1 (*EDS1*) gene (Xie et al., 2011). phyB serves as the upstream of elongated hypocotyl 5 (HY5) and participates in HY5 accumulation under RL (Osterlund et al., 2000). HY5, a basic leucine zipper (bZIP) transcription factor, is the first known positive regulator of light signaling under various wavelengths of light, including RL, far-RL, blue light, and UV-B (Oyama et al., 1997; Osterlund et al., 2000; Ulm et al., 2004). HY5 directly binds the G-box (CACGTG) DNA sequence of downstream genes, including light, ROS, and hormone-responsive genes (Lee et al., 2007; Shi et al., 2011; Chen et al., 2013; Abbas et al., 2014). HY5 positively regulates Pchlide synthesis, ROS production, and cell death in the light during seedling greening (Chen et al., 2013). However, limited information is known whether HY5 is involved in modulating ROS and SA signaling to promote PCD and enhance plant tolerance in response to RL.

Lesion simulating disease 1 (LSD1) encodes a small C_2_C_2_ zinc finger protein and acts as a negative regulator of PCD (Dietrich et al., 1997). LSD1 participates in controlling the redox signals generated from the PQ pool in the chloroplast and suppresses cell death under EL and RL conditions. LSD1 and EDS1 elicit opposite effects on ROS and SA-dependent PCD under UVC and cold stress (Muhlenbock et al., 2008; Huang et al., 2010; Wituszynska et al., 2013, 2015). The *lsd1* mutant shows an increase in the *EDS1* expression but displays a decrease in signal response (SR) family gene transcript levels in response to light stress (Wituszynska et al., 2013). These results suggest that the signal response 1 (SR1) protein combines with the DNA sequence (ACGCGT) of the *EDS1* promoter to suppress gene expression; the SR1 protein is necessary to repress SA-dependent defense (Du et al., 2009). However, the EDS1 gene sequence contains a G-box domain, which is possibly combined by HY5. A possible mechanism is that LSD1 suppresses the *EDS1* expression by upregulating *SR1*, but HY5 promotes the *EDS1* expression under RL.

LSD1 activity requires three C_2_C_2_-type zinc finger domains, and LSD1 is also a redox-sensitive protein (Dietrich et al., 1997). Previous studies suggested that the zinc finger protein often functions as a redox sensor, and redox targets are thiols in Zn/S-coordination center (Junming et al., 2014). After oxidizing treatment is administered, the zinc finger protein releases zinc and subsequently converts into oxidized conformation (dimerization state) to change activity (Hwang et al., 2001; Tsao and Su, 2001; Derong et al., 2005; Ungureanu et al., 2012). RL likely causes the more oxidized status of the cell and then inhibits LSD1 activity by changing conformation, which does not interact with other proteins. *Arabidopsis* basic leucine zipper transcription factor 10 (AtbZIP10) and LSD1 regulate antagonistically oxidative stress-induced PCD (Kaminaka et al., 2006). *Arabidopsis* metacaspase 1 (AtMC1) interacts with LSD1 and then blocks AtMC1-dependent PCD (Coll et al., 2010). GSH-induced LITAF domain protein (GILP) and CATALASEs (CAT1, CAT2, and CAT3) interact with LSD1 to negatively regulate hypersensitive cell death (He et al., 2011; Li et al., 2013).

In this study, LSD1 and HY5 performed opposite roles in regulating RL-triggered PCD associated with ROS and SA production. Unexpectedly, LSD1 conformation and activity were controlled by the change in PQ pool under RL. However, phyB interacted with HY5 and contributed to RL response. Our results demonstrated that LSD1 and HY5 antagonistically modulated the EDS1-dependent ROS and SA signaling; thus, PCD was mediated in response to RL.

## Materials and methods

### Plant materials, growth, light conditions

Arabidopsis ecotype Columbia-0 (Col-0), *lsd1-2* (Coll et al., 2010), *hy5-215* (Osterlund et al., 2000), *hy5-ox-YFP* (Oravecz et al., 2006), *phyb*, *phyB-ox-YFP* (Wang et al., 2010; Zhao et al., 2014), *eds1-2* (Wang et al., 2011) were sterilized and grown in soil culture with 16/8 h light/dark cycle (100 nm μmol photons m^−2^·s^−1^) and 54% relative humidity at 22°C. Four-week old plants were used for experiments. Arabidopsis rosettes were fully exposed to excess white light (EL, 1500 μmol m^−2^ s^−1^; 6 h) or (RL, 120 μmol photons m^−2^s^−1^; 6 h, 650 nm) supplied by light-emitting diode panels (Photon System Inst.). The above light conditions provided similar energy at the indicated spectral regions. Heat emission from the light source was insignificant. Plants were also sprayed with or exposed to a 30–40 mL drop of DCMU (8 μM) applied to the leaf 2–3 h before RL treatment as described (Friedman et al., 2012).

### Trypan blue staining

Four-week leaves were exposed to RL for the indicated period of time before staining. Trypan blue staining was performed as described (Muhlenbock et al., 2008). After staining, all samples were mounted on slides and photographed with a stereo microscope at 6 and 15-fold magnification.

### Electrolyte leakage analysis

Cell death was quantified by ion leakage from whole rosettes. Four-week-old plants treated with RL, DCMU, and DCMU + RL were harvested. If the leaves were big enough to be cored, leaf disks were removed (7 mm diameter), floated in water for 30 min and subsequently transferred to tubes containing 6 mL distilled water. Conductivity of the solution was detected with an Orion Conductivity Meter at the indicated time points. For each measurement, we used six leaf disks or 40–50 mg fresh weight, which equaled 4–6 leaves. The entire experiment was performed three times (Coll et al., 2010).

### Fluorescence imaging of ROS and hydrogen peroxide quantification

ROS fluorescence determination was performed, which the leaves in the dark for 1 h with 10 μM 2′7′-dichlorofluorescin diacetate (H_2_DCFDA) and oberving the stain by the Zeiss LSM 510. Hydrogen peroxide was quantified using previous method (Muhlenbock et al., 2008).

### SA quantifications

The total SA was extracted by previous method (Xing et al., 2013). The extracting total SA was detected by high performance liquid chtomatoraphy (HPLC) using fluorescence detectors: excitation wavelength is 294 nm and emission wavelength is 426 nm.

### Determination of glutathione

Glutathione levels were detected through the 5, 5′-dithiobis (2-nitrobenzoic acid)-recycling enzymatic method. The extracted samples were divided in half for assay of total glutathione (GSH + GSSG) and for GSSG alone as described previously (Ge et al., 2007). The absorbance change at 412 nm was measured, and the glutathione concentration was evaluated by comparison with a standard calibration curve.

### Catalase activity

CAT activity previously described and was measured spectrophotometrically by monitoring H_2_O_2_ decrease at 240 nm (Mateo et al., 2004).

### RNA extraction and qRT-PCR analysis

Total RNAs were extracted from detached Arabidopsis leaves using Trizol according to the supplier's recommendations. The integrity of the total RNA extracts was tested by 1% agarose gel electrophoresis. RNA concentrations and purities were measured spectrophotometrically using OD_260_/OD_280_ and OD_260_/OD_230_ ratios. First-strand cDNA was synthesized with the SuperScript II First-Strand Synthesis System for qRT-PCR (Invitrogen). qRT-PCR was performed using the Roche Light Cycler TM 2.0 Real-time Detection System. The expression of target gene was normalized relative to the housekeeping gene *ACTIN2* (Li et al., 2012). The primers are presented *LSD1* (5′-TGTCAAACTACGAACCTTGTGC-3′ and 5′-TTGATCTGCGCAACCTGA-3′); *HY5* (5′-ATGCAGGAACAAGCGACT-3′ and 5′-TCAAAGGCTTGCATCAGC-3′); *EDS1*(5′-CGAAGGGGACATAGATTGGA-3′and5′-ATGTACGGCCCTGTGTCTTC-3′); *PAD4* (5′-TATGGTCGACGCTGCCATAC-3′ and 5′-ATAGAAGCCAAAGTGCGGTG-3′); *NPR1* (5′-CCGCCGCTAAGAAGGAGAAA-3′and 5′-GCCAAAACAGTCACAACCGA-3′); *ICS1* (Du et al., 2009); *PAL*(Xing et al., 2013) and *PR1*(Zhao et al., 2014). The results were calculated using the 2^−ΔΔCT^ method. Preliminary statistics were performed using the Roche Light Cycler system and the significance of differences was analyzed in SPSS 15.0.

### Protein extraction and western blot analysis

For protein extraction, 0.4 g treated 14-day-old seedlings were ground to powder in liquid nitrogen and dissolved in extraction buffer [50 mM Tris–HCl, pH 6.8, 50 mM DTT, 4% (w/v) SDS, 10% (v/v) glycerol, 1% (w/v) polyvinylpolypyrrolidone (PVPP), 5 mM PMSF]. After centrifugation at 12,000 g for 20 min twice at 4°C, the supernatants were transferred into new tubes, quickly stored at −80 °C until analyzed. The protein content was determined by the BCA Protein Assay Kit (TIANGEN, China). Extracts containing 40 mg protein were separated by 12% sodium dodecyl sulfate–polyacrylamide gel electrophoresis (SDS–PAGE) and transferred to nitrocellulose membranes. The membranes were blocked with TBST (10 mM Tris–HCl, pH 7.4, 150 mM NaCl, 0.1% Tween-20) containing 5% non-fat milk for 1 h and then incubated with LSD1 antibody (Agrisera), HY5 antibody (Osterlund et al., 2000). After washing three times, the membranes were blocked with secondary antibody anti-rabbit IRDyeTM800 (Rockland Immunochemicals, Gilbertsville, PA, USA). Proteins were detected by using Odyssey two-color infrared imaging system (Li-Cor, Inc., Lincoln, NE, USA).

### Co-immunoprecipitation assay

Co-immunoprecipitation assay was performed as described previously (Zhao et al., 2014) with some modifications. Total proteins were extracted from plants in an extraction buffer [50 mM Tris-HCl, pH 7.5–8.0, 100 mM NaCl, 1% NP-40, 0.5% sodium deoxycholate, 0.1% SDS, 1 mM EDTA, 1 mM sodium or thovanadate, 50 mM sodium fluoride, 1 mM PMSF, containing protease inhibitor cocktail (Roche)]. Proteins extracts were inoculated with antibody for 4 h, and then protein A beads were added. After incubation overnight at 4°C, the beads were centrifuged and washed four times with a wash buffer (PBS, pH 7.4). The immunoprecipitated proteins were detected by SDS-PAGE gel with YFP antibody or LSD1 antibody (Agrisera). HY5 protein was detected with a rabbit polyclonal HY5 antibody (Osterlund et al., 2000) after immunoprecipitation with YFP and HY5 antibody (Zhao et al., 2014).

### Chromatin immunoprecipitation (ChIP)

For the ChIP analysis, 1.5 g fresh leaves were crosslinked with 10 mL of 1% formaldehyde under vacuum infiltration conditions. ChIP assays were performed as described previously (Vani et al., 2007) with a minor modification of the three wash buffers: Low wash buffer (150 mM NaCl, 0.1% SDS, 1% Triton X-100, 2 mM EDTA, 20 mM Tris-HCl, pH 8), High Salt Wash buffer (500 mM NaCl, 0.1% SDS, 1% Triton X-100, 2 mM EDTA, 20 mM Tris-HCl, pH 8), LiCl Wash Buffer (0.25 M LiCl, 1% v/v NP-40, 1% w/v sodium deoxycholate, 1 mM EDTA, 10 mM Tris-HCl, pH 8). The amount of each precipitated DNA fragment was determined by semi-quantitative PCR and qRT-PCR using to amplify the sequences with G-box regions contained in the *EDS1* gene promoter (from −221 to −101) (5′-AAAACCGACACGTGGAAAGC-3′ and 5′-CCCCATCATGAGACCATTTCA-3′) and the sequences without G-box regions (from −688 to −571) (5′-CGAACGCAAAAACGGACCAG-3′ and 5′-CTTATCCGGAAAGTAAACCGGA-3′).

### Statistical analysis

Data were subjected to analyzing by Duncan's multi- ple range tests (DMRTs; *p* < 0.05) and significance by analysis of variance (ANOVA) using SPSS12.0 software for Windows (Zhao et al., 2014).

## Results

### Excess light promotes programmed cell death regulated by LSD1 and HY5, red light is a major inducer

**Figure 1:**
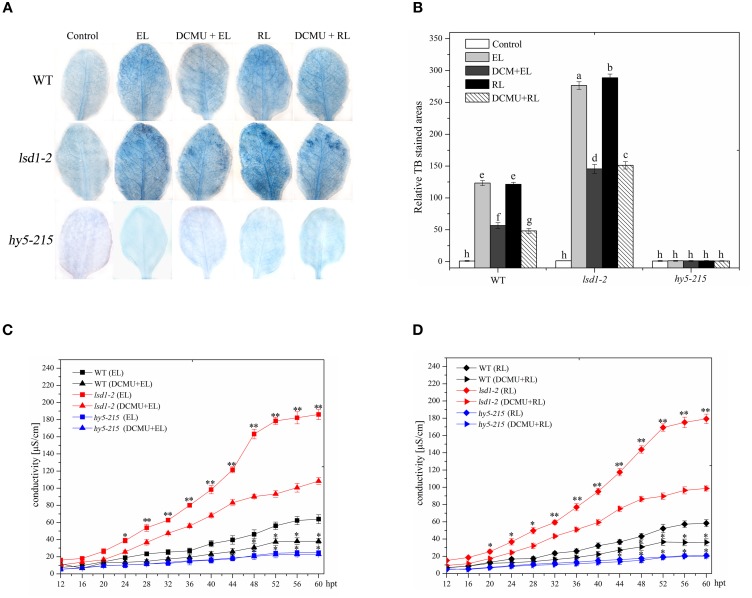
**Plant PCD in response to RL is regulated by LSD1, HY5 and the redox status of PQ pool. (A)** Trypan blue staining of four-week-old leaves of wild type(WT), *lsd1-2* mutant, and *hy5-215* mutant, which were exposed to normal light (control, 100 μmol photons m^−2^s^−1^), excess white light (EL, 1500 μmol m^−2^ s^−1^; 6 h) or excess red light (RL, 120 μmol m^−2^ s^−1^; 660–680 nm; 6 h) excess red light (RL; 120 μmol photons m^−2^s^−1^; 6 h), and were pre-sprayed 8 μM DCMU under normal light for 3 h and then exposed to EL or RL for 6 h (DCMU + RL). **(B)** Areas of TB-stained foliar tissues in WT, *lsd1-2*, and *hy5-215* mutant as described in **(A)**. Different letters indicate statistically significant differences between treatments (Duncan's multiple range test: *P* < 0.05). Values represent means ± SD of three independent experiments. **(C,D)** Ion leakage of WT, *lsd1-2* mutant, and *hy5-215* mutant.

In *Arabidopsis* wild-type leaves, excess white light or excess RL caused PCD, as indicated by trypan blue staining and ion leakage detection results; by contrast, the extent of cell death in response to RL was similar to EL irradiation. The *lsd1-2* mutant showed a more prominent increase in PCD than the WT under EL and RL treatments. However, a significant decrease in PCD was observed in the *hy5-215* mutant (Figure 1). The redox state of the PQ pool was involved in regulating EL-induced PCD and light acclimatory responses (Muhlenbock et al., 2008). EL and RL maintain the redox state of PQ to a more reduced state to promote PCD, whereas DCMU inhibits this process and maintains the redox state of PQ to a more oxidized state (Vandewalle et al., 2007; Muhlenbock et al., 2008; Busch, 2014). The *lsd1-2* mutant and WT treated with DCMU under ambient light for 4 h and exposed to EL or RL showed a significant decrease in PCD compared with that treated with RL alone; by contrast, the *hy5-215* mutant leaves of the same treatment were slightly different (Figure 1). These results indicated that LSD1 and HY5 acted as negative and positive regulators, respectively, in EL-induced PCD; RL was a major inducer. The redox status of PQ is implicated in RL-dependent PCD.

### RL-induced ROS and SA accumulations are controlled by LSD1 and HY5

**Figure 2:**
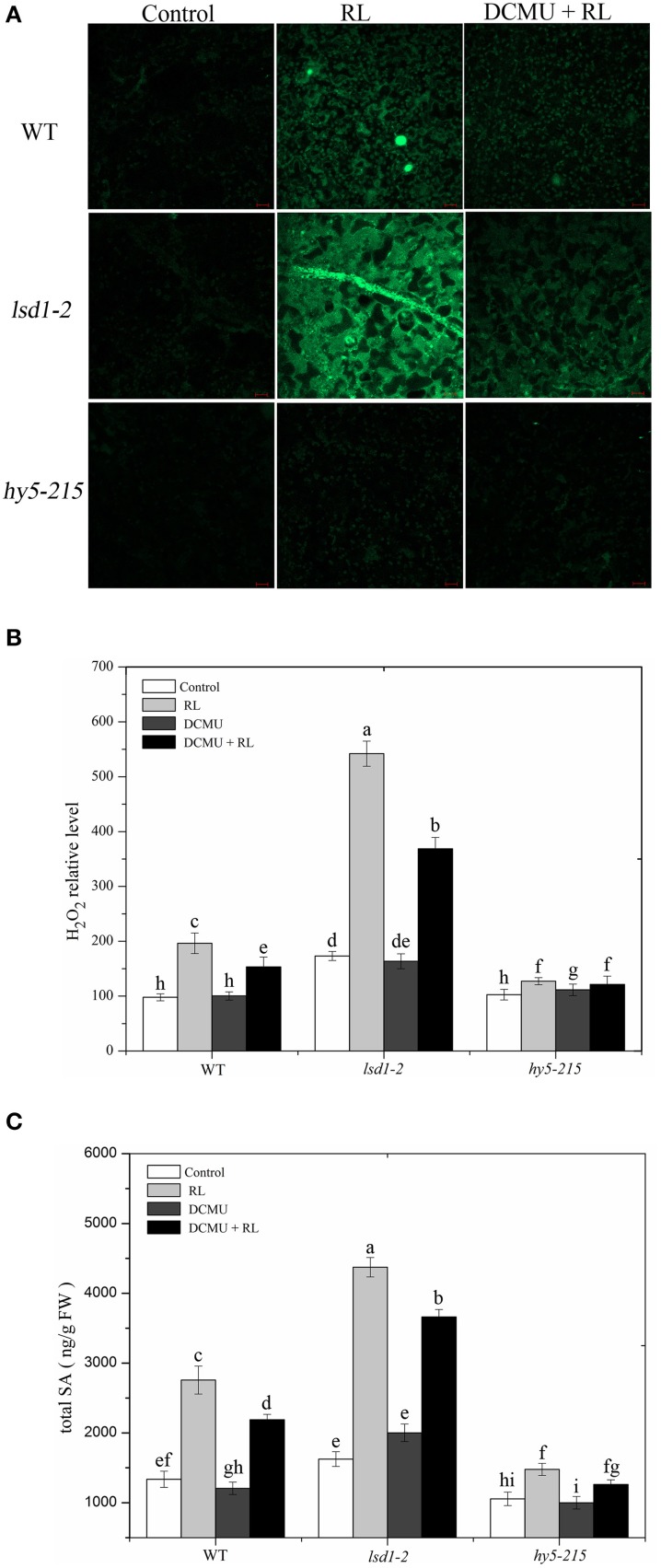
**Plant cellular ROS/SA homeostasis modulated by LSD1, HY5, and the redox status of PQ pool under red light. (A)** H_2_DCFDA fluorescence (green) indicates ROS in four-week-old leaves of WT, *lsd1-2* mutant, and *hy5-215* mutant, which were exposed to control, RL for 6 h, and DCMU + RL. **(B)** The cellular H_2_O_2_ levels by the quantitative measurement in four-week-old leaves of WT, *lsd1-2* mutant, and *hy5-215* mutant exposed to different treatment, including control, RL, DCMU, and DCMU +RL. **(C)** The cellular SA levels were detected in WT, *lsd1-2* mutant, and *hy5-215* mutant, the four-week old leaves were exposed to control, RL, DCMU, and DCMU+ RL. Data are means ± SD of three experiments. Different letters indicate statistically significant differences between treatments (Duncan's multiple range test: *P* < 0.05).

A previous study substantiated that EL-induced cell death is regulated by subsequent changes in cellular ROS and hormonal homeostasis, not by changes in ROS alone (Mackintosh et al., 1989). RL leads to ROS and SA accumulation, and therefore promotes PCD (Mateo et al., 2004; Muhlenbock et al., 2008; Szechynska-Hebda et al., 2010). H_2_O_2_ and SA concentrations in WT and two mutant (*lsd1-2* and *hy5-215*) plants were measured in the present study. The exposure of WT to RL for 6 h resulted in a significant increase in H_2_O_2_ and SA concentrations (Figure 2). At the same time, H_2_O_2_ and SA concentrations were higher in the RL-treated *lsd1-2* mutant than in WT; the production of H_2_O_2_ and SA of the *hy5-215* mutant was prevented compared with that of WT (Figure 2). This result indicated that LSD1 could suppress the accumulation of H_2_O_2_ and SA. However, HY5 could promote accumulation in RL-induced PCD. H_2_O_2_ and SA concentrations were remarkably lower in the *lsd1-2* mutant and WT pre-treated with DCMU and then treated with RL than in plants treated with RL alone; by contrast, H_2_O_2_ and SA concentrations in the *hy5-215* mutant were weakly decreased. The results suggested that LSD1 and HY5 elicited an antagonistic effect on RL-induced ROS release, SA accumulation, and PCD, which were partially dependent on the redox status of the PQ pool (Figures 2A–C). Our results suggested that LSD1 and HY5 had antagonistic effect on RL-induced ROS burst, SA accumulation, and consequently PCD, which were partially dependent on the redox status of PQ pool.

### RL changes the cellular redox status and subsequently affects LSD1 conformation and activity

Many redox-regulated proteins, such as Hsp33 and SP1, use a cysteine-coordinating zinc center as a redox sensor. Upon exposure to oxidized condition, cysteines are quickly oxidized and zinc is released (Maret, 2006). LSD1 is a novel zinc finger protein that contains three zinc finger domains; this protein is also a redox-regulated protein. To determine whether RL can cause cellular redox state to change, we analyzed the content of reduced glutathione (GSH) and the ratio of reduced glutathione: total glutathione ([GSH]:[(GSH) + (GSSG)]) in RL-treated WT. The results showed that RL caused an increase in GSH levels within 6 h. GSH levels were maximal at 12 h after RL treatment was administered and decreased thereafter (Supplementary Figure 1A). A significant decrease in the redox status of the glutathione pool after 12 h indicated that oxidative stress was induced by RL irradiation (Supplementary Figure 1B). The GSH levels decreased when WT was pre-treated with DCMU and then exposed to RL for 12 h, but the (GSH):([GSH] + [GSSG]) ratio significantly increased compared with that of the 12 h RL treatment (Supplementary Figures 1C,D). The GSH levels and the (GSH):([GSH] + [GSSG]) ratio remained unchanged in the chloroplast, whereas the cytoplasmic (GSH):([GSH] + [GSSG]) ratio was altered and similar to the trend of the total cell (Supplementary Figure 1). The results demonstrated that RL disturbed the cellular PQ pool balance and subsequently resulted in a more oxidized environment in the cytoplasm.

**Figure 3:**
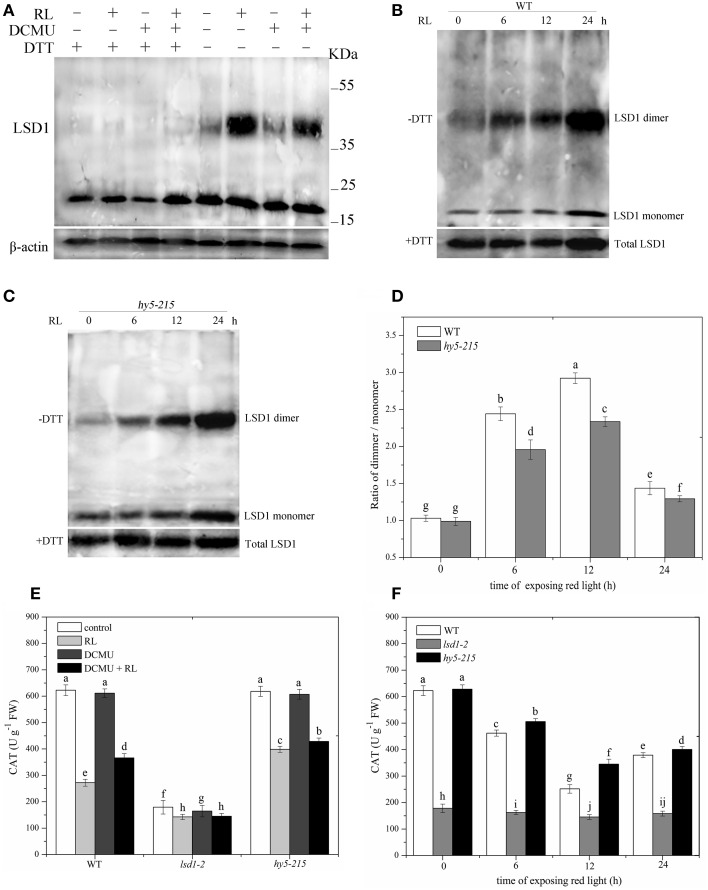
**Conformation of LSD1 was changed by immunoprecipitation assay and LSD1 activity was indirectly measured under RL. (A)** Total protein extracted with (+) or without (–) DTT (50 mM) in the extraction buffer from control, RL, DCMU, and DCMU + RL treated WT was subjected to immunoprecipitation analysis, both dimmer, and monomer LSD1 protein were detected. **(B)** Total protein was extracted from WT leaves at times after RL treatment and analyzed by an immunoprecipitation assay. **(C)** The LSD1 conformation was detected in *hy5-215* mutant. **(D)** The ratio of LSD1 dimer/monomer was analyzed quantitatively. **(E)** The CAT activity was measured in WT, *lsd1-2* mutant, and *hy5-215* mutant, the four-week old leaves were exposed to control, RL, DCMU and DCMU+ RL for 6 h. **(F)** The CAT activity of WT, *lsd1-2* mutant, and *hy5-215* mutant was detected at times after RL treatment. Different letters indicate statistically significant differences between treatments (Duncan's multiple range test: *P* < 0.05). Values represent means ± SD of three independent replicates.

The reducing agent DTT, included in the protein extraction solution, can modify protein conformation and function by reducing protein cysteine residues. Protein samples extracted with or without DTT were then subjected to immunoblot analysis in WT to investigate the specific conformation of the LSD1 protein. The addition of DTT to all samples reduced all of the LSD1 proteins to the monomeric form. Without DTT, the LSD1 protein was present at a higher molecular weight (dimer) in RL treatment but not in the control group (Figure 3A). Under RL, dimer and monomer of the LSD1 protein increased; by contrast, the ratio of dimer/monomer decreased. The dimer of LSD1 of plants subjected to DCMU pre-treatment and RL treatment was significantly decreased compared with those subjected to RL treatment alone (Figure 3A). To explore whether HY5 is involved in regulating the LSD1 conformation, we detected LSD1 conformation in the *hy5-215* mutant without DTT. The results showed that the ratio of dimer/monomer of LSD1 in the *hy5-215* mutant was slightly lower than that in WT after RL treatment was administered (Figures 3B–D). The RL-induced PQ pool change combined with HY5 modulated the LSD1 protein conformation change, and the redox status of the PQ pool played a major role.

To analyze LSD1 activity, we indirectly examined CAT activity because the active LSD1 positively regulates CAT activity by interacting with CAT (Li et al., 2013). The CAT activity was lower in RL-treated WT than in the control group. The CAT activity of plants pre-treated with DCMU and exposed to RL was significantly enhanced compared with that of plants treated with RL alone. The *lsd1-2* mutant also displayed the strong inhibition of the CAT activity, particularly in the RL treatment. However, the CAT activity showed a weak reduction in the *hy5-215* mutant compared with that of WT (Figures 3E,F). The results showed that RL induced a more oxidized GSH pool, which mainly depended on the redox status of the PQ pool change, and caused the monomer of LSD1 to convert into dimer and suppress the LSD1 activity. HY5 weakly influenced the LSD1 conformation change and activity.

### HY5 interacts with phyB in cell death induced by RL

**Figure 4:**
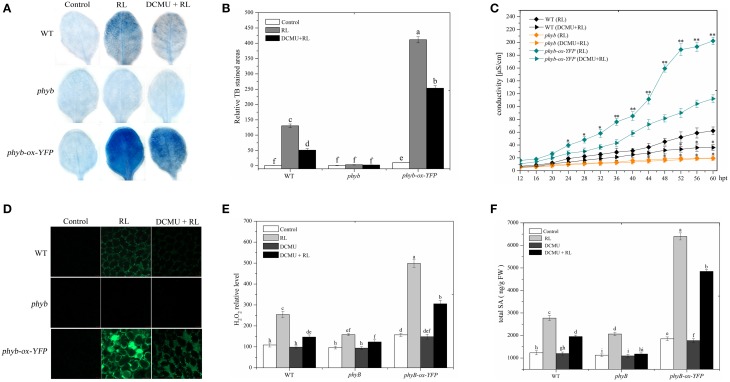
**phyB is involved in regulating RL-induced PCD associated with ROS and SA production. (A)** Trypan blue staining of four-week-old leaves of WT, *phyB* mutant, and *phyB-ox-YFP* under control, RL, and DCMU + RL treatment. **(B)** Areas of TB-stained foliar tissues in WT, *phyB* mutant, and *phyB-ox-YFP*as described in **(A)**. **(C)** Relative ion leakage in WT, *phyB* mutant, and *phyB-ox-YFP* after RL or DCMU + RL. Asterisks indicate significant differences between the wild type and the mutants (Student's paired *t*-test: ^*^P < 0.05, ^**^P < 0.01). **(D)** H_2_DCFDA fluorescence (green) indicates ROS in leaves of WT, *phyB* mutant, and *phyB-ox-YFP* exposed to control, RL for 6 h, and DCMU + RL. **(E)** Measurement of ROS levels of WT, *phyB* mutant, and *phyB-ox-YFP* under control, RL, DCMU, and DCMU + RL conditions. **(F)** Detection of SA levels. Different letters indicate statistically significant differences between treatments (Duncan's multiple range test: *P* < 0.05). Values represent means ± SD of three independent replicates.

**Figure 5:**
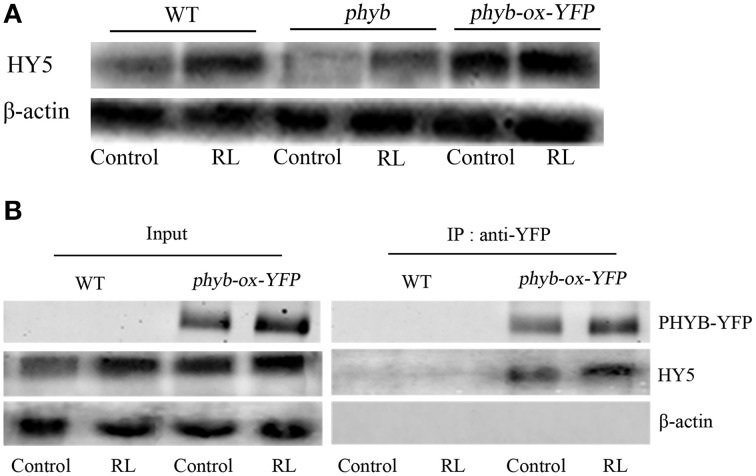
**HY5 interacts with phyB and promoted HY5 accumulation under RL. (A)** The western blot analysis the HY5 of protein levels in WT, *phyB* mutant, and *phyB-ox-YFP* transgentic plant under untreated and RL treatment. **(B)** *In vivo* coimmunoprecipitation assay between phyB and HY5, WT and *phyB-ox-YFP* plant was exposed to control and RL. After precipitation with the anti-phyB-YFP antibody, proteins were immunoblotted with anti-HY5 or anti-YFP antibodies.

phyB is a specific RL-response factor, which positively modulates cell death and ROS signaling (Li et al., 2011; Chen et al., 2013). In this study, the *phyB* mutant significantly inhibited PCD and decreased ROS and SA concentrations; by contrast, *phyB-ox-YFP* significantly enhanced PCD and increased ROS and SA levels compared with WT exposed to RL (Figure 4). The *phyB* mutant and *phyB-ox-YFP* were also exposed to DCMU and RL. PCD and ROS/SA concentrations in *phyB-ox-YFP* decreased compared with those in *phyB-ox-YFP* subjected to RL treatment alone; by contrast, PCD and ROS/SA concentrations in the *phyB* mutant weakly decreased (Figure 4). It has also been found that phyB acted as upstream of HY5 and positively regulates HY5 (Osterlund et al., 2000). The mutation of *phyB* inhibited HY5 protein accumulation. However, the overexpressed *phyB* line increased the HY5 protein concentration compared with WT (Figure 5A). However, whether phyB promotes HY5 protein accumulation through the interaction of phyB with HY5 Co-immunoprecipitation assays were performed using proteins isolated from *phyB-ox-YFP* transgenic plants or WT to ensure that this interaction occurred *in vivo*. Figure 5B showed that the YFP antibody could immunoprecipitate the HY5 fusion protein, as detected by the HY5 antibody, under control and RL conditions. The result showed HY5 could interact with phyB under control and RL. The data suggested that phyB-mediated HY5 accumulation may rely on the interaction of phyB and HY5 under the RL-induced PCD.

### EDS1 is required for RL induced-cell death and its expression is modulated antagonistically by LSD1 and HY5

**Figure 6:**
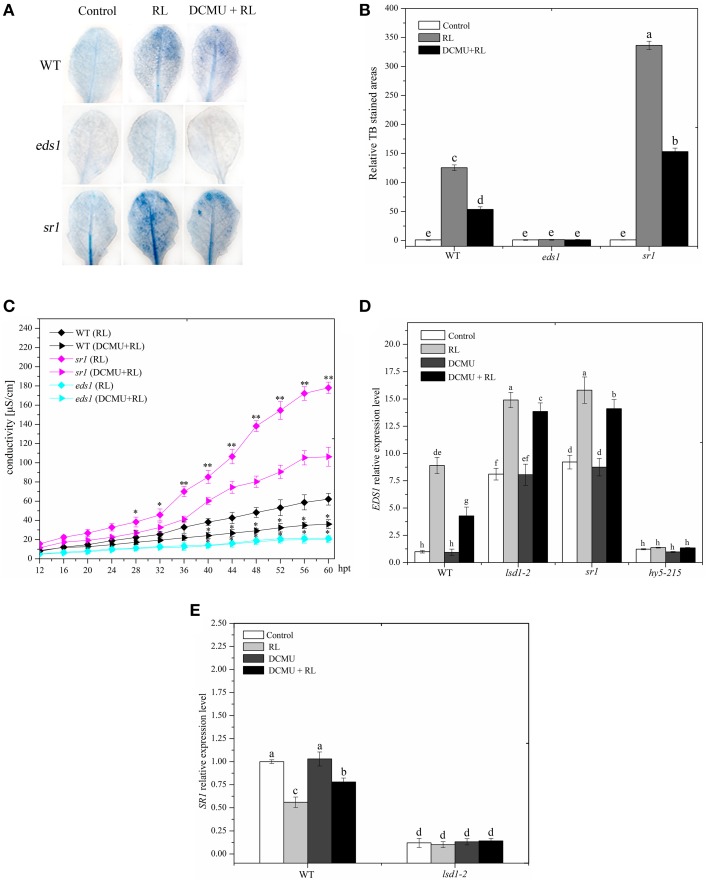
**EDS1 is in response to RL-induced PCD and its expression is controlled by LSD1 and HY5. (A)** Trypan blue staining of four-week-old leaves of WT, *eds1* and *sr1* mutant after control, RL, and DCMU + RL treatment. **(B)** Areas of TB-stained foliar tissues in WT, *eds1* and *sr1* mutant as described in **(A)**. **(C)** Relative ion leakage was detected and showed the extent of cell death. Asterisks indicate significant differences between the wild type and the mutants (Student's paired *t*-test: ^*^P < 0.05, ^**^P < 0.01). **(D)** qRT-PCR showing the relative expression of *EDS1* gene in WT, *hy5-215,lsd1-2* and *sr1* mutant exposed to control, RL, DCMU and DCMU + RL. **(E)** qRT-PCR determination of *SR1* mRNA levels in WT and *lsd1-2* mutant exposed to control, RL, DCMU, and DCMU + RL. Each data is the mean ± SD of three independent replicates. Different letters indicate statistically significant differences between treatments (Duncan's multiple range test: *P* < 0.05).

EDS1 is necessary to upregulate ROS and ET production in EL stress and caused the onset of cell death in the *lsd1* mutant (Mateo et al., 2004). PCD was severely suppressed in the *eds1* mutant compared with that in WT after RL treatment was administered (Figures 6A–C). The *eds1* mutant also showed decreased ROS and SA concentrations compared with WT exposed to RL (Supplementary Figure 3). RL induced an increase in the *EDS1* expression levels in WT (Figure 6C). The result indicated that EDS1 is required for RL-induced ROS and SA signaling and PCD.

LSD1 negatively regulates EDS1-dependent PCD during EL (Muhlenbock et al., 2008). The *EDS1* expression levels were increased in the *lsd1-2* mutant compared with those in WT exposed to RL, indicating that LSD1 inhibited the *EDS1* expression (Figure 6D). This previous study also showed that phyA and phyB could positively modulate the EDS1-dependent SA signaling by improving *EDS1* expression (Xie et al., 2011). PhyB may promote the EDS1 expression by upregulating HY5. To determine whether HY5 positively regulates *EDS1* expression, we performed qRT-PCR analysis of the *hy5-215* mutant and WT after RL treatment was administered. Our results showed that the EDS1 expression levels strongly decreased in the *hy5-215* mutant than in WT regardless of RL treatment (Figure 6D). These data demonstrated that HY5 could enhance the *EDS1* expression. The EDS1 expression in plants pre-treated with DCMU and then treated with RL decreased compared with that in WT treated with RL only; however, no evident change occurred in *lsd1-2* and *hy5-215* mutants (Figure 6D). These results indicated that the PQ pool was also involved in the *EDS1* expression in response to RL. Thus, LSD1 and HY5 antagonistically modulated the *EDS1* expression during RL-induced cell death.

### LSD1 inhibits EDS1 gene expression by contributing to SR1 expression

SR1 directly binds to promoter of *EDS1* gene, and consequently suppressed *EDS1* gene expression (Du et al., 2009). Recent study has showed that LSD1 positively regulates *SR* family expression (Wituszynska et al., 2013); SR1 prevents cell death, biotic defense responses, and RL response (Galon et al., 2008, 2010). The *sr1* mutant displayed a strong increase in cell death and ROS/SA production compared with WT after RL treatment was administered (Figures 6A–C and Supplementary Figure 3). However, no difference was observed between *sr1* and *lsd1-2* mutants (Figures 1, 2, 6). The*EDS1* expression levels were enhanced in *lsd1-2* and *sr1* mutants compared with those in WT; by contrast, the *EDS1* expression levels were similar between *lsd1-2* and *sr1* mutants regardless of RL treatment (Figure 6D). Cell death and ROS/SA generation were partly suppressed and *EDS1* expression was decreased when the *sr1* mutant was pre-treated with DCMU and then exposed to RL compared with that treated with RL alone (Figures 6A–C). Therefore, SR1 and LSD1 possibly prevented RL-induced PCD by inhibiting *EDS1* expression.

LSD1 may be involved in regulating *SR1* gene expression. *SR1* expression levels decreased in the *lsd1-2* mutant compared with WT. *SR1* expression levels also decreased in WT exposed to RL than in the control group but were similar to the *lsd1-2* mutant between the control group and the RL-treated group (Figure 6E). The *SR1* expression also decreased in WT exposed to DCMU and then to RL compared with that exposed to RL alone. However, no difference was observed in the *lsd1-2* mutant (Figure 6E). These results demonstrated that LSD1 inhibited the *EDS1* expression by upregulating *SR1* expression, but RL inhibited SR1 gene expression by inducing PQ pool to change.

### HY5 directly binds the promoter of EDS1 gene to promote its expression

**Figure 7:**
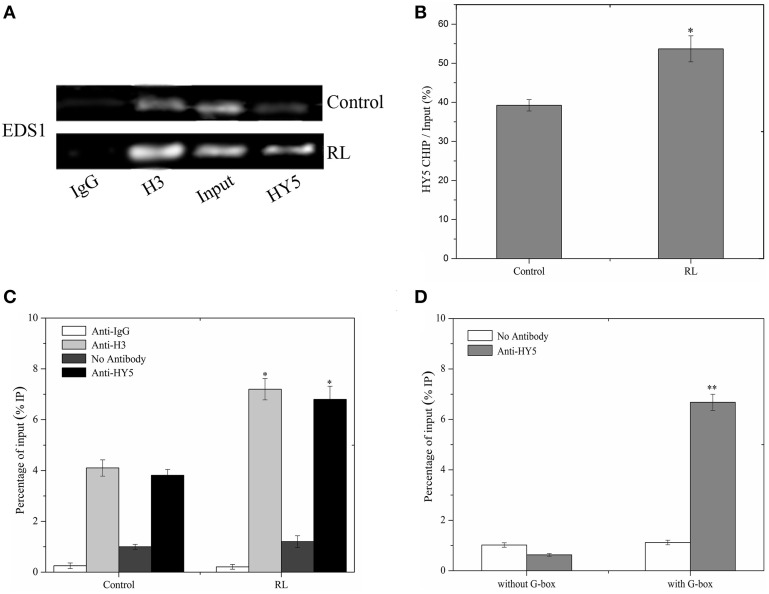
**HY5 binds directly to the promoter of *EDS1*. (A)** A ChIP assay was performed with specific antibodies for immunoprecipitation, and purified DNA samples were analyzed by RT-PCR assay. Immunoprecipitation was carried out with normal rabbit IgG (negative control, lane 1), anti-acetyl-histone H3 (positive control, lane 2), and anti-HY5-YFP serum (lane 4). As a control for detecting the efficiency of the ChIP assay, “input DNA” samples (lane3) without immunoprecipitation, were processed in parallel. **(B)** Quantitative analysis of *EDS1* gene with anti HY5-YFP was shown in **(B)** with Image J software about **(A)**. **(C)** ChIP-qPCR analysis of the *EDS1* promoter sequence under normal and RL conditions. **(D)** ChIP-qPCR assays were performed to analyse the binding of HY5 protein to the G-box motifs of *EDS1* promoter. Error bars indicate ±SD values. Statistical analysis was performed with Student's paired *t*-test: ^*^P < 0.05, ^**^P < 0.01 in compared with control.

W This study showed that HY5 positively mediated the *EDS1* transcript expression induced by RL (Figure 7C). HY5 acted as a positive transcription factor and directly bound a G-box (CACGTG) DNA sequence element in many light-regulated genes (Lee et al., 2007). It is interesting that promoter of *EDS1* also contains G-box, which may be the binding region of HY5. To investigate whether HY5 could combine with the *EDS1* promoter, a chromatin immunoprecipitation (ChIP) assay was performed followed by RT-PCR and analyses on *hy5-ox-YFP* transgenic plants with or without RL treatment. Our data showed that ChIP with either anti-acetyl-histone 3 or anti-HY5-YFP antibody detected the EDS1 promoter amplification products (Figure 7A, lane 2 and 4). However, almost no products were observed in ChIP with anti-rabbit IgG (Figure 7A, lane 1). As a control setup of ChIP efficiency, the input DNA samples without immunoprecipitation displayed a bright lane (Figure 7A, lane 3 and Figure 7B). ChIP–qPCR analysis also illustrated that HY5 could interact with G-box within the *EDS1* promoter regardless of RL (Figure 7C). As shown in Figure 7D, HY5 strongly binds to the *EDS1* promoter that encompasses the G-box motifs; by contrast, no interaction was observed between HY5 and EDS1 promoter without the G-box motifs. These results suggested that HY5 could bind to the EDS1 promoter and induce HY5 expression under RL.

## Discussion

### LSD1 and HY5 antagonistically regulate PCD associated with ROS and SA production triggered by RL

In this study, the *lsd1-2* mutant displays an enhanced cell death, whereas the *hy5-215* mutant shows a suppressed cell death in response to RL (Figure 1), as indicated by trypan blue staining and ion leakage measurement; this result suggested that LSD1 and HY5 serve as negative and positive regulators of RL-mediated PCD, respectively. RL also induces ROS release and SA accumulation. The *lsd1-2* mutant also accumulates higher ROS and SA concentrations, whereas the *hy5-215* mutant accumulates lower ROS and SA concentrations under RL; this result indicated that LSD1 and HY5 play different roles in regulating ROS and SA signaling in response to RL (Figure 2).

LSD1 prevents cell death and enhances light acclimation; LSD1 is also involved in modulating ROS and hormone signaling (Mateo et al., 2004; Muhlenbock et al., 2008). Light-dependent cell death is related to H_2_O_2_ and SA accumulation in the *lsd1* mutant (Li et al., 2013). LSD1 positively modulates superoxide dismutase and *CAT* gene expression and CAT activities; therefore, LSD1 controls cellular ROS production (Kliebenstein et al., 1999; Mateo et al., 2004; Li et al., 2013). LSD1 negatively regulates RL-induced PCD by controlling ROS/SA signaling. Under EL stress, the onsets of PCD in the *lsd1* mutant partly depends on the redox status of the PQ pool, which is required for EDS1 (Muhlenbock et al., 2008). The data showed that the *lsd1-2* mutant and WT pre-treated with DCMU partly inhibit PCD and ROS/SA production exposed to RL (Figures 1, 2). The change in the redox status of the PQ pool may act upstream of LSD1 to regulate PCD in response to RL.

HY5, as a transcription factor that binds to the promoter ROS-response genes, promotes ROS production and cell death during seedling greening under long-term light conditions (Chen et al., 2013). HY5 is also involved in modulating the signaling of various hormones, including auxin, cytokinin, brassinosteroids, and gibberellins (Vandenbussche et al., 2007; Alabadi and Blazquez, 2008; Shi et al., 2011). HY5 integrates ROS with SA signaling to regulate RL response (Figure 2). HY5 is downstream of phyB in light signaling pathways (Osterlund et al., 2000). The *phyAphyB* mutant reduces cell death and blocks ROS and SA accumulation (Griebel and Zeier, 2008). The phyB plays a crucial roles in red light signaling (Neff et al., 2000). phyB also positively mediates PCD and ROS and SA accumulation under RL (Figure 4). HY5 may promote RL-triggered PCD by phyB signaling pathway and act independent of the PQ pool in the chloroplast.

LSD1 and HY5 had an antagonistic effect on SA accumulation by regulating SA-related gene expression in response to RL. It was observed that RL induced the expression of *EDS1*, *non-expressor of pathogenesis-relatedgenes1 (NPR1)* and *isochorismate synthase1 (ICS1)*, but didn't influence the expression of *phytoalexin deficient4 (PAD4)* and *phenylalanine ammonia-lyase (PAL)*. Meanwhile, *EDS1* expression was only and antagonistically controlled by LSD1 and HY5 (Figure 6D and Supplementary Figure 5), which indicated that *EDS1* played a key role in the regulation of RL-dependent SA signaling pathways.

### RL prevents LSD1 activity by changing the LSD1 conformation

LSD1 that contains three zinc finger domains acts as a redox-sensor protein (Dietrich et al., 1997). LSD1 and Hsp33 also contain similar amino acid sequences of zinc finger domain, which is C-x-x-C zinc finger type. This sequence is sensitive to oxidized conditions. A previous study confirmed that Hsp33 releases zinc ion from the zinc finger domain, and the inactive monomer is converted into the active dimer under oxidizing conditions (Graf and Jakob, 2002). In our study, RL resulted in a more oxidized GSH pool in the cytoplasm by disrupting the PQ pool. Thus, RL caused a more oxidized cellular environment. Subsequently, the reduced monomeric LSD1 may transform into the oxidized dimeric LSD1 by removing zinc; the active LSD1 was converted into the inactive LSD1, as indicated by the CAT activity (Figure 3). The active LSD1 interacts with CATs (CAT1, CAT2, and CAT3) and positively controls CAT activity (Li et al., 2013). Therefore, LSD1 activity may be dependent on monomer conformation and complete zinc finger, which interact with other proteins.

Although LSD1 and HY5 play different roles in the PCD process in response to RL, these factors did not influence each other in terms of expression and protein levels (Supplementary Figure 2). The LSD1 protein contains three zinc finger domains and HY5 is a bZIP family protein (Oyama et al., 1997). AtbZIP10 interacts with LSD1 in the cytoplasm and suppresses AtbZIP10 activity by blocking the AtbZIP10 translocation to the nucleus (Kaminaka et al., 2006). To determine whether LSD1 interacted with HY5, Co-immunoprecipitation assays showed that LSD1 did not interact with HY5 (Supplementary Figure 2A). The localization of subcelluar LSD1 remained unchanged under RL treatment, and HY5 was only localized in the nucleus (Supplementary Figures 2E,F). Considering that LSD1 is not directly related to HY5, we investigated LSD1 conformation and activity in the *hy5-215* mutant exposed to RL. The result indicated that HY5 partially changed LSD1 protein conformation and repressed activity (Figure 4). The PQ pool change combined with HY5 modulated ROS levels and disrupted the cellular redox status, resulting in changes in the LSD1 structure and activity in RL-induced PCD. However, the function of RL-induced PQ pool change was more important than that of HY5.

### LSD1 suppresses EDS1 expression by upregulating SR1 expression

LSD1 negatively modulates EDS1-dependent cell death under long-term light, UVC, cold stress, and pathogen stress exposure (Muhlenbock et al., 2008; Huang et al., 2010; Wituszynska et al., 2013, 2015). The *eds1* mutant displayed an inhibition of cell death and a decrease in ROS/SA production after RL treatment; this result indicated that EDS1 is required for PCD in response to RL (Figures 6A–C and Supplementary Figure 3). The *EDS1* expression was significantly increased in response to RL (Figure 6D). Previous data showed that *EDS1* expression levels are increased in the *lsd1* mutant (Wituszynska et al., 2013). Our data implied that LSD1 could suppress *EDS1* expression under RL condition. SR1 also directly combines with the *EDS1* promoter and inhibits the *EDS1* expression (Du et al., 2009). The *sr1* mutant showed an enhanced *EDS1* expression and exhibited a remarkable increase in cell death, as well as higher ROS/SA concentrations. This result was similar to that observed in the *lsd1-2* mutant exposed RL (Figures 1, 6 and Supplementary Figure 3). LSD1 repressed the *EDS1* expression by upregulating the *SR1* expression. The LSD1 protein may act as a transcription regulator to modulate the *SR1* gene expression; however, the underlying molecular mechanism requires further research. This study showed that the *SR1* expression was dependent on the change in PQ pool exposed to RL. Thus, RL may repress LSD1 activity and inhibit *SR1* expression by altering the redox status of the PQ pool.

### HY5 interacts with phyB and promotes EDS1 expression during RL-induced PCD

phyB specifically functions in response to RL and enhances plant defense under RL (Zhao et al., 2014). Under RL, the inactive phyB turns into a photo-active phyB and is transferred from the cytoplasm to the nucleus (Nagatani, 2004). The interaction between phyB and COP1 rapidly inhibits the COP1 activity under RL (Luo et al., 2014). In darkness, HY5 interacts with COP1 and is degraded via the 26S proteasome pathway. However, RL promoted HY5 accumulation (Osterlund et al., 2000). The HY5 protein was decreased in the *phyB* mutant, but the HY5 protein was increased in the *phyB-ox-YFP* transgenic plant under RL treatment (Figure 4F). Meanwhile, it was found that phyB more significantly interacted with HY5 under RL condition, and the effect may be because of the increase of HY5 protein (Figure 5B). This process possibly involves the activation of phyB induced by RL, and the activated phyB may interact with HY5 and promoted HY5 accumulation by inhibiting COP1 activity.

**Figure 8:**
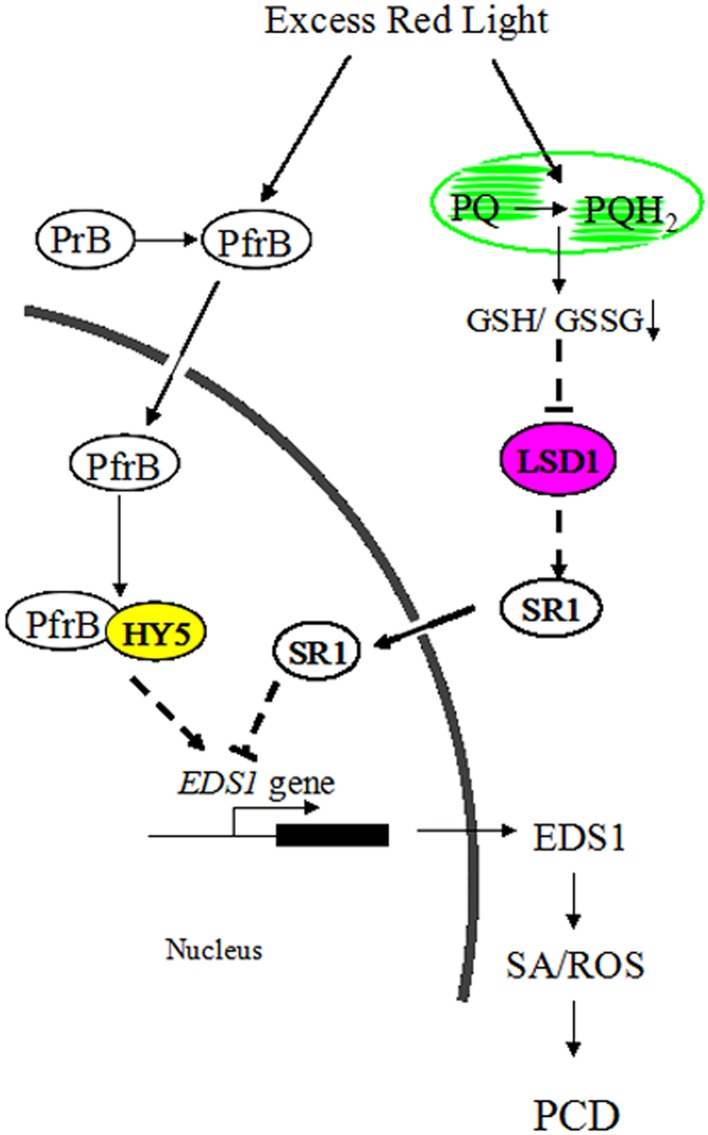
**A possible model for the roles of LSD1 and HY5 in antagonistically regulating PCD induced by RL in*Arabidopsis***.

The genes related to the SA-dependent defense pathway and the *EDS1* expression were downregulated in the *phyAphyBphyC* mutant (Xie et al., 2011). The results indicated that phyB and HY5 positively controlled the *EDS1* expression and induced PCD under RL (Figure 6D and Supplementary Figure 4). HY5 serves as a transcription factor, which combines with DNA sequence (G-box); thus, ROS-relative gene was regulated (Lee et al., 2007). The results showed that HY5 could more evidently bind to the G-box motif of the EDS1 gene promoter and positively regulate *EDS1* expression under RL (Figures 6D, 8). Due to RL-induced the accumulation of HY5, the abundance of HY5 might increase the chance to bind EDS1. Hence, phyB-mediated RL signaling is dependent on ROS and SA signaling modulated by HY5.

Although ROS signaling could be integrated into SA signaling to regulate adaptive responses in plants, components connecting ROS with SA pathways remain unknown (Mittler et al., 2011). In this study, light signaling could interact with ROS and SA signaling, and LSD1 and HY5 represent a key convergence point between these signaling pathways. RL resulted in a more oxidized environment by altering PQ pool, where the active monomer of LSD1 was converted into the inactive dimer of LSD1. However, the inactive LSD1 did not contribute to the *SR1* expression and relieved inhibition of the EDS1 expression. RL also positively modulated HY5 by activating phyB. Subsequently, HY5 interacted with phyB and enhanced the EDS1 expression by binding to the G-box of the EDS1 promoter. As a result, plants induced ROS release, SA accumulation, and cell death in response to RL (Figure 8). Indeed, the molecular mechanism of RL signaling with other cellular pathways provides novel insights into these resulting processes.

### Conflict of interest statement

The authors declare that the research was conducted in the absence of any commercial or financial relationships that could be construed as a potential conflict of interest.

## Supplementary material

Supplementary Figure 1 **RL-induced change of cellular redox status**.

Supplementary Figure 2 **LSD1 and HY5 don't influence on each other under RL**.

Supplementary Figure 3 **EDS1 and SR1 influence on ROS and SA production in response to RL**.

Supplementary Figure 4 **The phyB positively regulates *EDS1* expression**.

Supplementary Figure 5 **LSD1 and HY5 have different effects on SA-related genes expression under RL**.
