# Supplementary figures and images for: LSD1 and HY5 antagonistically regulate red light induced-programmed cell death in Arabidopsis
Source: Front Plant Sci. 2015 May 5;6:292. doi: 10.3389/fpls.2015.00292 (PMC4419654; doi:10.3389/fpls.2015.00292)

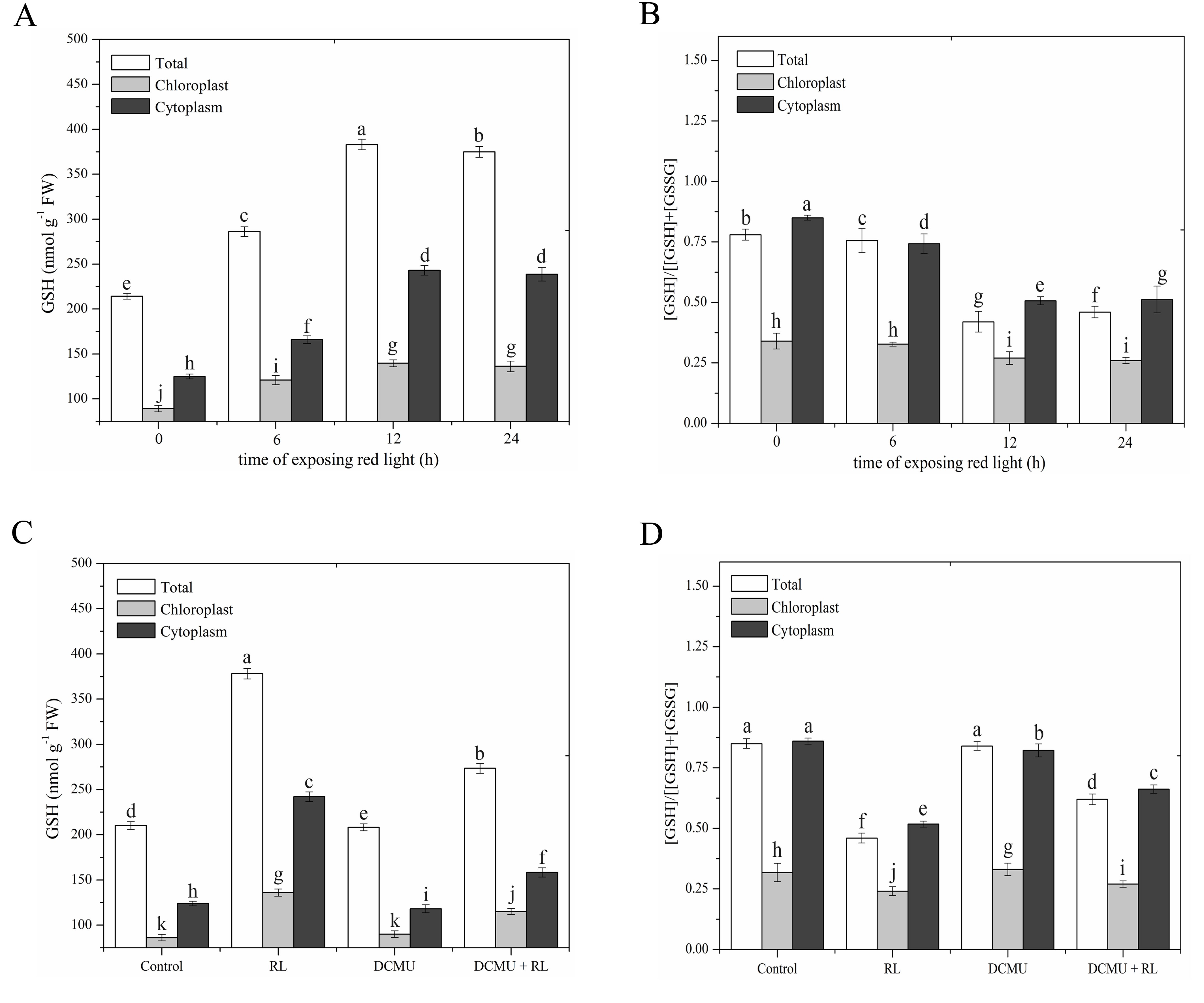

Supplement: Supplementary Figure 1 — RL-induced change of cellular redox status. [file Image1.TIF]

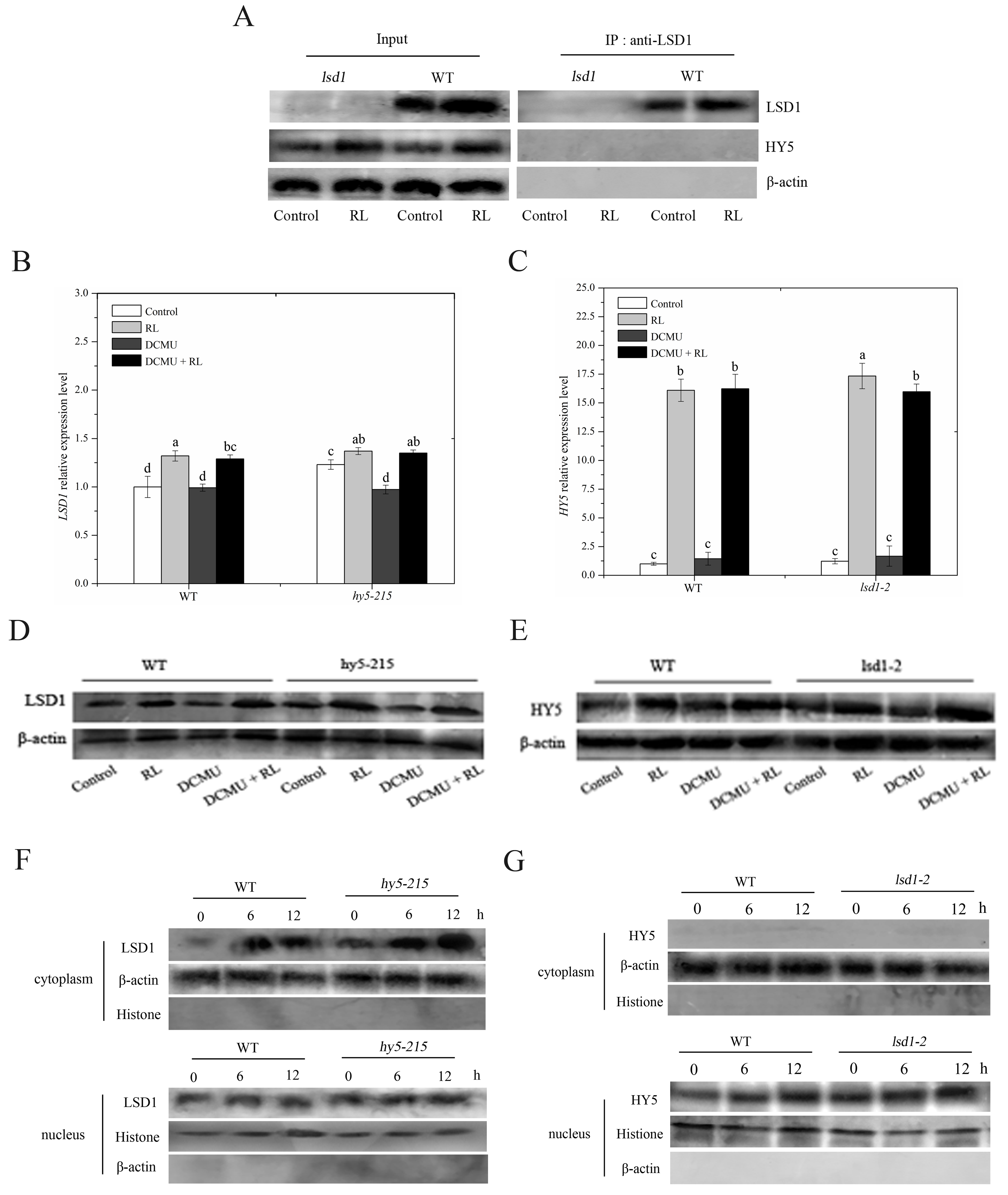

Supplement: Supplementary Figure 2 — LSD1 and HY5 don't influence on each other under RL. [file Image2.TIF]

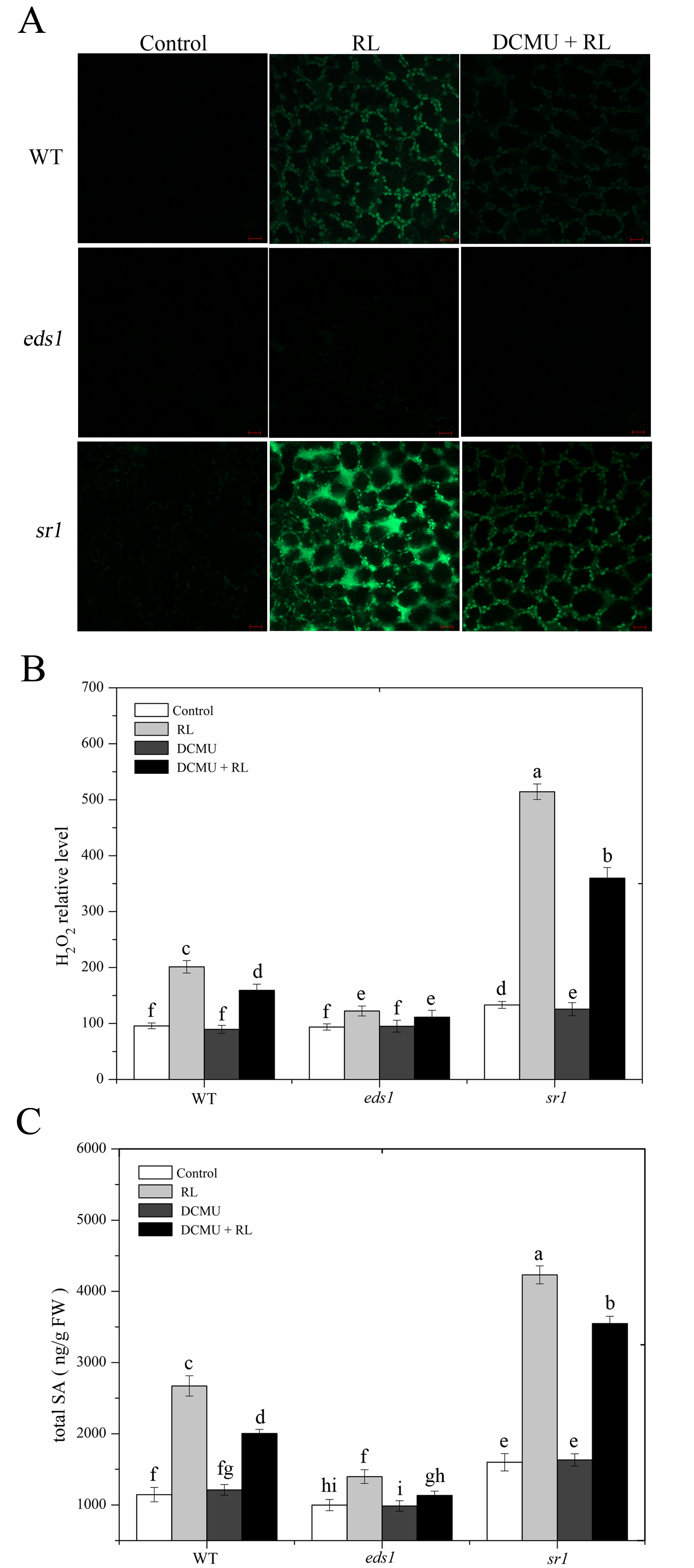

Supplement: Supplementary Figure 3 — EDS1 and SR1 influence on ROS and SA production in response to RL. [file Image3.TIF]

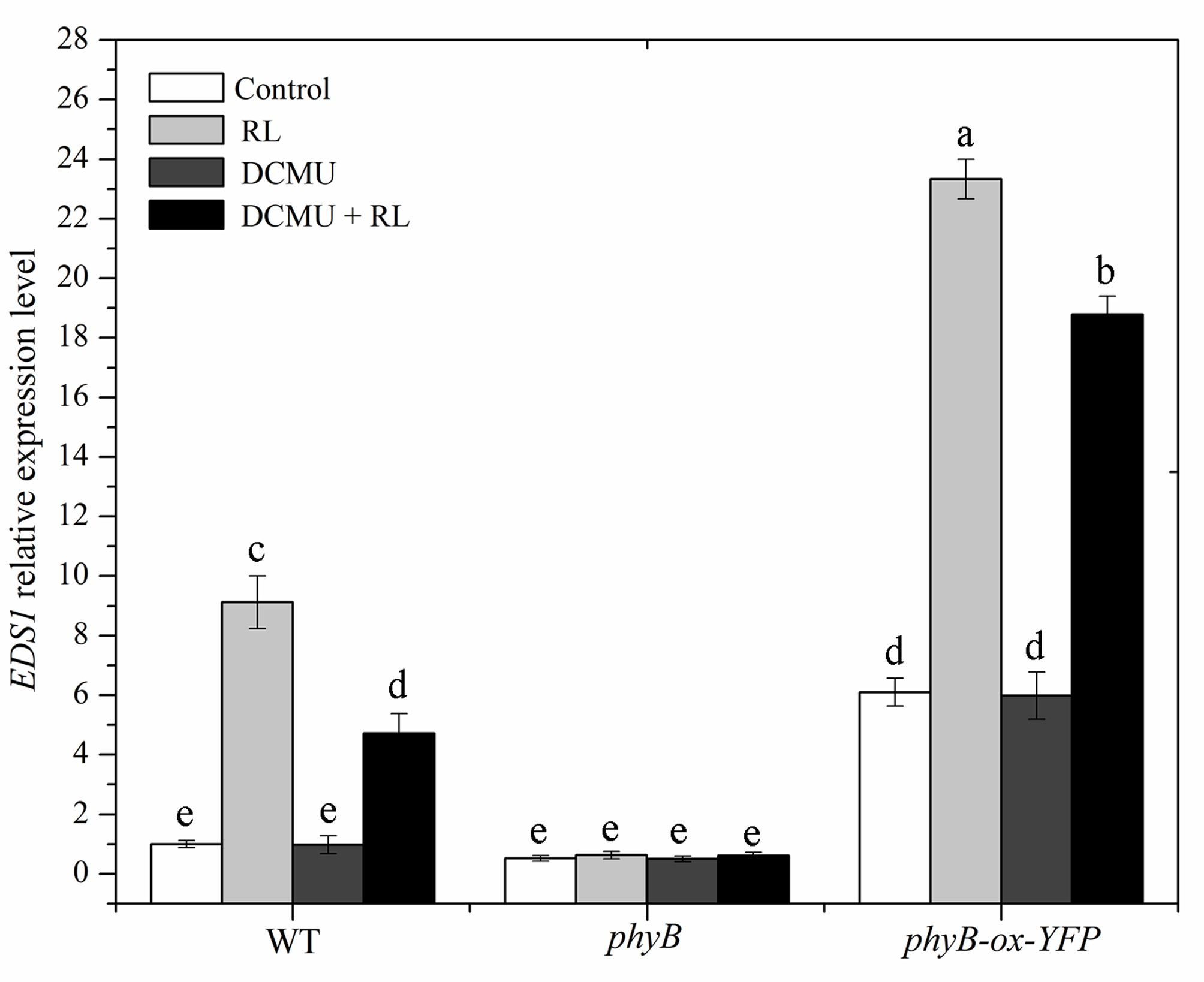

Supplement: Supplementary Figure 4 — The phyB positively regulates EDS1 expression. [file Image4.TIF]

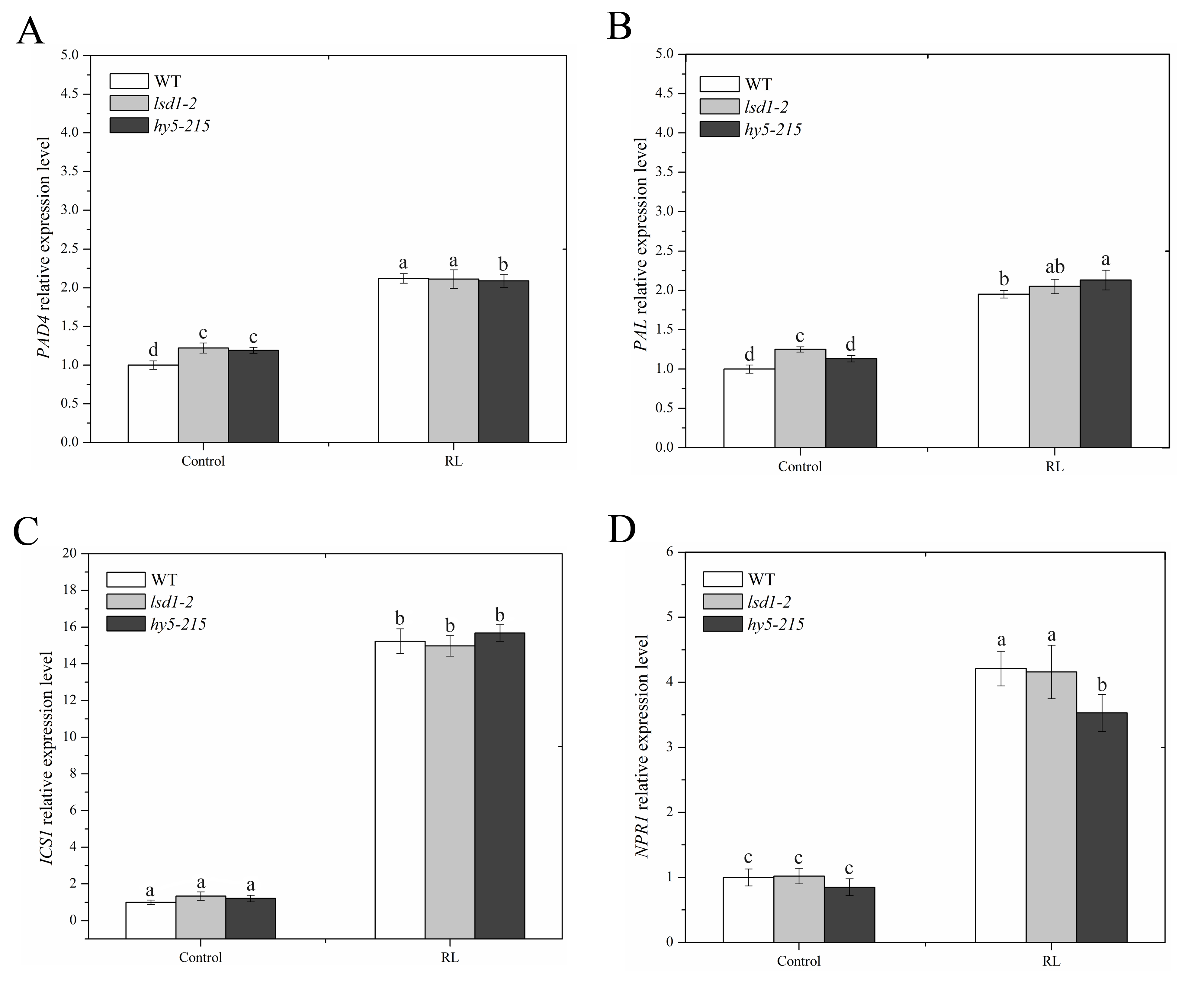

Supplement: Supplementary Figure 5 — LSD1 and HY5 have different effects on SA-related genes expression under RL. [file Image5.TIF]
